# A novel and more efficient biosynthesis approach for human insulin production in *Escherichia coli* (*E. coli*)

**DOI:** 10.1186/s13568-020-00969-w

**Published:** 2020-03-10

**Authors:** Kamini Govender, Tricia Naicker, Johnson Lin, Sooraj Baijnath, Anil Amichund Chuturgoon, Naeem Sheik Abdul, Taskeen Docrat, Hendrik Gerhardus Kruger, Thavendran Govender

**Affiliations:** 1 Catalysis and Peptide Research Unit (CPRU), School of Health Sciences, University of KwaZulu-Natal, E-block, 6th Floor, Room E1-06-016, Westville Campus, Durban, South Africa; 2 School of Life Sciences, University of KwaZulu-Natal, Durban, South Africa; 3 School of Laboratory Medicine and Medical Sciences, College of Health Sciences, University of KwaZulu-Natal, Durban, South Africa; 4 Department of Chemistry, University of Zululand, Private Bag X1001, Kwadlangezwa, 3886 South Africa

**Keywords:** Biosynthesis of human insulin, Diabetes, *E*. *coli*

## Abstract

Insulin has captured researchers’ attention worldwide. There is a rapid global rise in the number of diabetic patients, which increases the demand for insulin. Current methods of insulin production are expensive and time-consuming. A PCR-based strategy was employed for the cloning and verification of human insulin. The human insulin protein was then overexpressed in *E. coli* on a laboratory scale. Thereafter, optimisation of human insulin expression was conducted. The yield of human insulin produced was approximately 520.92 (mg/L), located in the intracellular fraction. Human insulin was detected using the MALDI-TOF-MS and LC–MS methods. The crude biosynthesised protein sequence was verified using protein sequencing, which had a 100% similarity to the human insulin sequence. The biological activity of human insulin was tested in vitro using a MTT assay, which revealed that the crude biosynthesised human insulin displayed a similar degree of efficacy to the standard human insulin. This study eliminated the use of affinity tags since an untagged pET21b expression vector was employed. Tedious protein renaturation, inclusion body recovery steps, and the expensive enzymatic cleavage of the C-peptide of insulin were eliminated, thereby making this method of biosynthesising human insulin a novel and more efficient method.

## Introduction

In 2018, it was estimated that 405.6 million people suffered from Type 2 diabetes, and this number is projected to increase to approximately 510.8 million by the year 2030. Based on these estimates, the global usage of insulin is estimated to rise from 516.1 million vials (1000 IU) to 633.7 million vials in 2030 (Basu et al. 2019). The increase in diabetic patients, coupled with the development of oral and inhalation methods of insulin delivery, requires considerable amounts of insulin. The current producers of insulin would not be able to cope with the rapid demand of affordable insulin as a result of high production costs and production capacity limitations (Baeshen et al. 2014).

Insulin has an essential role in glucose homeostasis (Ahmad 2004). It is produced by the beta cells of the pancreas and is one of the main anabolic hormones in the human body (Voet and Voet 2011). It is synthesised by beta cells in the pancreas and has a fundamental role in fat and carbohydrate metabolism (Ahmad 2004). Human insulin is a 51 amino acid (aa) polypeptide. It contains two polypeptide chains. The A chain has 21 amino acids and the B chain contains 30 amino acids. Insulin has three disulfide bonds. Two of the disulfide bonds interlink the A and B chains, whereas the third one is an intra A chain bond (Ahmad 2004; Vajo et al. 2001). Insulin was first discovered in 1921 by Charles Best and Frederick Banting (Banting and Best 1922). Prior to 1982, insulin was extracted from the pancreas of animals such as bovines and porcine (Beals et al. 2013; Mollerup et al. 2009).

The biotechnological industry can, possibly, develop suitable innovations to curb the shortage in the supply of human insulin. Novel innovations in methods of insulin production and purification have the potential to significantly advance the pharmaceutical biotechnology industry. The use of recombinant deoxyribonucleic acid (DNA) technology allowed for a direct method of biosynthesising human insulin, which did not require animal-derived pancreatic tissue (Walsh 2005). In 1982, Novo Nordisk derived semi-synthetic human insulin, whereby porcine insulin was converted into human insulin enzymatically. Human insulin was biosynthesised by Eli Lilly using recombinant DNA technology in 1982. Two production methods were reported by Eli Lilly (Mollerup et al. 2009). The first method consisted of cloning the A and B chains of insulin separately in *E*. *coli*; thereafter, these two chains were isolated, purified, and chemically attached. Subsequently, the final reversed phase-high performance liquid chromatography (RP-HPLC) purification steps were conducted (Chance et al. 1981; Frank and Chance 1983; Mollerup et al. 2009). The second method of cloning recombinant insulin in *E*. *coli* consisted of the expression of proinsulin under a tryptophan promoter that had a methionine bond to proinsulin. This study cloned the A and B chains of human insulin together in *E*. *coli*. In 1988 Novo Nordisk launched the biosynthesis of human insulin using recombinant DNA technology (Mollerup et al. 2009). The proinsulin biosynthesis strategy of human insulin is the preferred method. However, this procedure results in the formation of inclusion bodies and requires the use of enzymes such as carboxypeptidase B to remove the C-peptide (Redwan et al. 2007; Zieliński et al. 2019) and the use of affinity tags (Redwan et al. 2007). The inclusion body formation requires additional steps to be taken, such as the renaturation of the protein (Redwan et al. 2007; Zieliński et al. 2019). This study aimed to create a novel and more efficient method for the biosynthesis of human insulin employing a polymerase chain reaction (PCR)-based strategy using the pET21b expression vector in *E*. *coli* for the optimisation of human insulin expression.

## Materials and methods

### Bacterial strains

The bacterial strains employed in this study were obtained from the School of Life Sciences, in the Discipline of Microbiology (University of KwaZulu-Natal, Westville Campus, South Africa). The pCMV6-XL5 plasmid integrated with the gene encoding for human proinsulin was procured from ORI Gene (United States of America (USA). Commercial human insulin protein was obtained from Sigma-Aldrich Inc (Germany) as a control in this study. All restriction enzymes were purchased from Thermo-Scientific, USA.

### Cloning of human insulin in *E*. *coli*

An amplification of human insulin was conducted using a PCR master mix according to the manufacturer’s instructions (Thermo-Scientific, USA). The pCMV6-XL5 plasmid was used as a DNA template with the following primers: (PGEX-BamHI-F: 5′-**GGA TCC** ATG GCC CTG TGG ATG CG-3′ and PGEX-XhoI-R: 5′-**CTC GAG** CTA GTT GCA GTA GTT CTC C-3). The PCR conditions were as follows: a denaturation at 95 °C for 30 s, followed by an annealing step at 61.2 °C for 30 s; thereafter an elongation step at 72 °C for 1 min, and lastly another elongation at 72 °C for 2 min. The PCR comprised 30 cycles. The PCR amplicons were subsequently visualised on a 1.5% agarose gel (Helling et al. 1974) and viewed using a Syngene G BOX gel documentation system (Vacutec, South Africa). The respective band was cut off using a scalpel under a UV trans-illuminator (UVP incorporated, USA). PCR products were then purified using the Zymoclean™ gel DNA recovery kit (ZYMO Research, USA), following the manufacturer’s instructions.

The pET21b vector DNA was isolated using a Zyppy™kit (ZYMO Research, USA), as per the manufacturer’s instructions. Thereafter, the PCR amplicons and the pET21b vector were digested with BamHI and XhoI, according to the manufacturer’s instructions (Thermo-Scientific, USA). Thereafter, the restricted PCR products were inserted into the pET21b vector using T4 DNA ligase (Thermo-Scientific, USA), according to the manufacturer’s instructions. The pET21b vector human proinsulin (designated as pET21b-hPin) was transformed in chemically competent *E. coli* BL21 (DE3) cells plasmid using the calcium chloride heat shock method (Inoue et al. 1990).

### The expression and isolation of the protein

The transformed *E. coli* BL21 (DE3) (Thermo-Scientific, USA) was verified using colony PCR. The positive clones were incubated in 10 mL of Luria-Bertani (LB) medium (1% bacto tryptone, 0.5% yeast extract, 1% NaCl) containing 50 μg/mL ampicillin and 34 μg/mL chloramphenicol (Merck, Germany) overnight at 37 °C at 180 revs per minute (rpm). One millilitre of an overnight culture was used to inoculate 100 mL of LB broth using the above method (Maseko et al. 2016; Volontè et al. 2011). The expression of human proinsulin was induced by the addition of 0.1 to 1 mM Isopropyl β-d thiogalactoside (IPTG) at an early exponential phase (OD_600_ 0.4–0.7).

The over-expressed protein was recovered following to a similar method adapted from Maseko et al. (2016). After overnight induction at 16 °C, the cultures were transferred to 50 mL Cell Star^®^ centrifuge tubes (Greiner Bio-One, Austria) and spun down at 8000 rpm for 15 min at 4 °C. The pellet was resuspended in 50 mM Tris–HCl (pH = 8). The crude protein extracts were sonicated for 5 to 10 min on ice until the cells were homogenised with a Sonic Ruptor 400 Ultrasonic homogeniser (Omni International, United States of America). Thereafter, the samples were centrifuged at 8000 rpm for approximately 15 min at 4 °C, and the supernatant was transferred to a clean centrifuge tube and stored at 4 °C. The proinsulin was converted to mature human insulin as a result of the autocatalytic cleavage of the C-peptide (Gooch 2011). Thereafter, the crude protein was concentrated using 3 kDa (kilo-Dalton) Amicon^®^ultra-size exclusion centrifugal filters (Merck, Germany), according to the manufacturer’s instructions.

### Detection of human insulin by matrix-assisted laser desorption ionization-time of flight-mass spectrometry (MALDI-TOF-MS)

The autoflex III smartbeam MALDI-TOF-MS (Bruker Daltonics, Germany) was used for the detection of the standard human insulin and biosynthesised human insulin samples. The MALDI-TOF spectra were obtained from linear mode, where by ions charged at a voltage of 20 kilovolts (kV) within a molecular mass detection range of 1500 to 7000 Daltons (Da) were employed. The instrument contained an ultraviolet nitrogen laser at 337 nanometres (nm). FlexControl version 3.4 build 119 software was used for the data acquisition and FlexAnalysis of the MALDI-TOF spectra. The target that was employed in the study was a ground steel target plate (Bruker Daltonics, Germany). The protein samples were diluted and spotted on the ground steel plate according to the manufacturer’s instructions (Bruker Daltonics, Germany). The matrix utilised in this study was alpha-cyano-4-hydroxycinnamic acid (CHCA or HCCA) (Bruker Daltonics, Germany). A standard curve was generated whereby standard human insulin was diluted to the required concentration using the HCCA matrix. The standard curve comprised of 0 ng/mL, 10 ng/mL, 50 ng/mL, 100 ng/mL, and 1000 ng/mL concentrations (refer to Additional file 1: Figure S4).

### Liquid chromatography-mass spectrometry (LC–MS)

The sample containing human insulin was further confirmed using the LC–MS-2020 (Shimadzu, Japan) with a YMC-Triart C18 column (150 mm × 4.6 mm internal diameter, pore size of 120 Å and a particle size of 5 μm) (YMC, Japan), coupled to a NM32LA nitrogen generator (Peak Scientific Instruments, United Kingdom). The MS spectra were obtained using the positive mode with a mass range of 200 to 1500 m/z. Mobile phase A was Millipore water (Millipore, USA) and mobile phase B was acetonitrile (Merck, Germany). Both these mobile phases contained 0.1% (v/v) formic acid as an ion-pairing agent. Flow rates of 1.0 mL/min were used with a total run time of 25 min. The gradient profile was from 5 to 95% acetonitrile in 7 min; and at 95% acetonitrile for a further 18 min. The column temperature was maintained at 40 °C. The nebuliser was set at 1.5 bar with a desolvation gas temperature of 250 °C and dry gas flow rates of 10 L/min.

### Protein sequencing of human insulin

An in-solution digestion of the protein samples were conducted at the Central Analytical Facility (Stellenbosch University, South Africa) on an Ultimate 3000 RSLC (Thermo Fisher Scientific, USA), coupling with a mass spectrometry (fusion mass spectrometer) (Thermo Fisher Scientific, USA) and an ionisation source (Nanospray Flex). The files generated from the mass spectrometer were imported onto the Proteome Discoverer v1.4. The procedure was conducted according to the manufacturer’s instructions (Thermo Fisher Scientific, USA), and analysed using an Amanda algorithm and Sequest. Thereafter, a concatenated database interrogation was conducted using the Uniprot P09211, concatenated with the contaminant protein database (cRAP). The peptides were validated using Target-Decoy PSM validator mode. The results were imported according to the manufacturer’s instructions using Scaffold 1.4.4 software (Proteome Software Inc, USA), and the identified peptides were validated with the peptide, Protein Prophet, as well as X!Tandem algorithms from the Scaffold 1.4.4 software (Proteome Software Inc, USA).

### Biological activity

The biological activity of the standard human insulin and the crude biosynthesised human insulin was conducted in vitro using a 3-(4, 5-dimethylthiazol-2-yl)-2, 5-diphenyl tetrazolium bromide (MTT) assay. A hepatocellular carcinoma (HepG2) (Highveld Biologicals, South Africa) cell line was used. The HepG2 cells were cultured according to a method similar that reported by Abdul et al. (2016). The cell culture reagents were obtained from Whitehead Scientific (South Africa). The standard human insulin and crude biosynthesised human insulin stocks were made in Eagle’s essential minimal medium (EMEM) (catologue number: 12-136F, Lonza, Switzerland) up to a concentration of 1 mg/mL. In the MTT assay, three treatment concentrations were used of the standard human insulin and crude biosynthesised human insulin: low (50 μg/mL), medium (100 μg/mL), and high (150 μg/mL).

The HepG2 cells were seeded into a 96-well plate (2 × 10^4^ cells/well, 24 h). Thereafter, the culture medium was removed, and cells were washed with PBS. The HepG2 cells were then treated with standard human insulin and crude biosynthesised human insulin hyperglycaemic (25 mM) media for 15 min (Chen et al. 2006). The treatments were removed by washing the cells in PBS, and 120 µL MTT (5 mg/mL, PBS) was added to each well. This was followed by an incubation of 4 h at 37 °C. The formazan crystals were solubilised by the addition of 100 µL/well DMSO. The optical densities (OD) were measured spectrophotometrically (BioTek uQuant, USA) at 570 nm with a reference wavelength of 690 nm. All experiments were conducted in triplicate.

The following formula was used to calculate cell viability (%):

$${\text{\% }}Cell\;viability = \frac{OD\;of\;treated\;cells}{OD\;of\;control\;cells} \times 100$$

### Statistical analysis

Two-way analysis of variance (ANOVA) was conducted. Analyses were performed using the statistical software package Graph pad in stat version 8.1.0 (325) 64 bit for Windows (Graph pad software, San Diego California).

## Results

### PCR amplification and purification of human proinsulin replicons

**Fig. 1:**
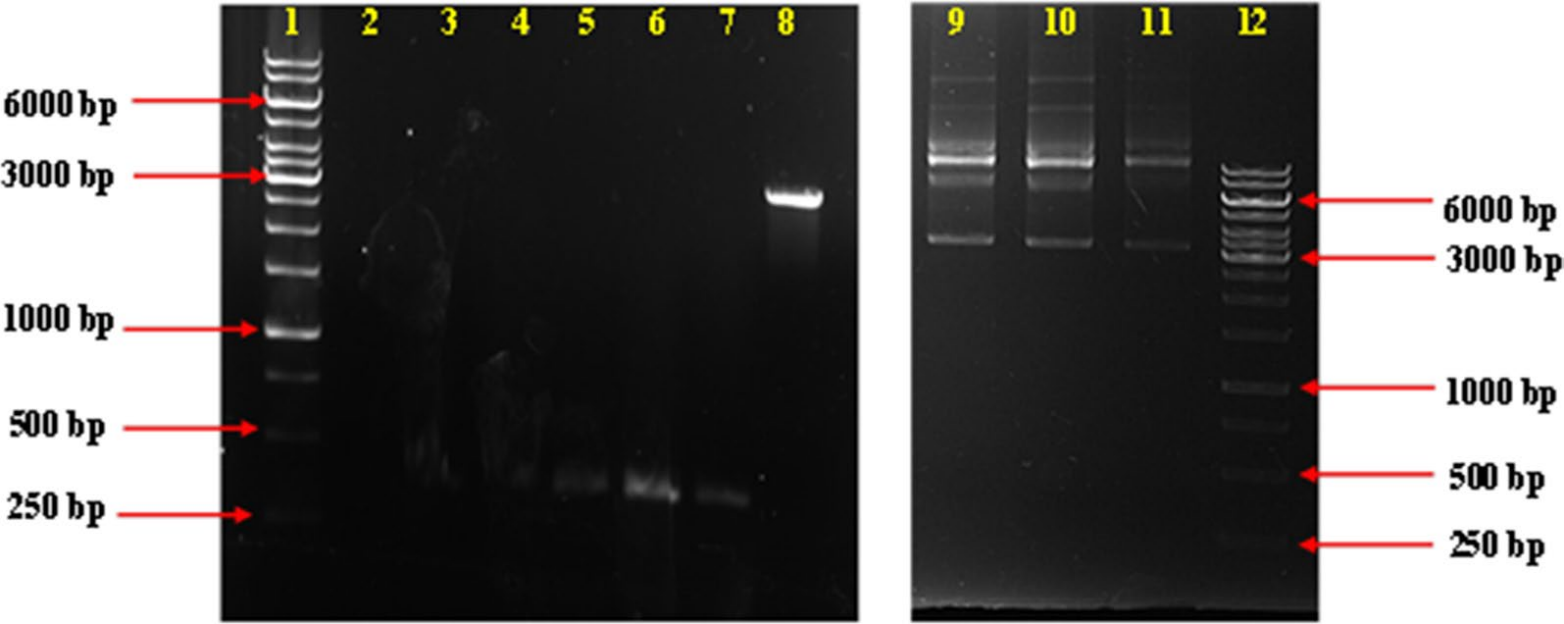
Images displaying the PCR amplicons of proinsulin DNA, purified vector DNA, and the integrated pET21b-hPin vector with the inserted proinsulin gene. Lanes 1 and 12 contain one kb molecular weight marker. Lanes 2 to 7 contains the PCR amplicons of proinsulin flanked with BamHI and XhoI restricted ends; Lane 8 contains the purified pET21b miniprep product; Lanes 9 to 11 contain the integrated pET21b-hPin vector miniprep products

A PCR amplification of human proinsulin was conducted using PCR primers PGEX-BamHI-F and PGEX-XhoI-R. The mass amplified and purified product corresponded to the expected amplicon size of 345 bp (Additional file 1: Figure S1a, lane 2). The human proinsulin replicons were inserted into pET21b. The pET21b-hPin plasmid (Fig. 1) was then transformed successfully into *E. coli* BL21 (DE3) cells, which were confirmed by colony PCR (345 bp; Additional file 1: Figure S1b).

### Detection and optimisation of expression of human insulin using MALDI-TOF-MS

**Fig. 2:**
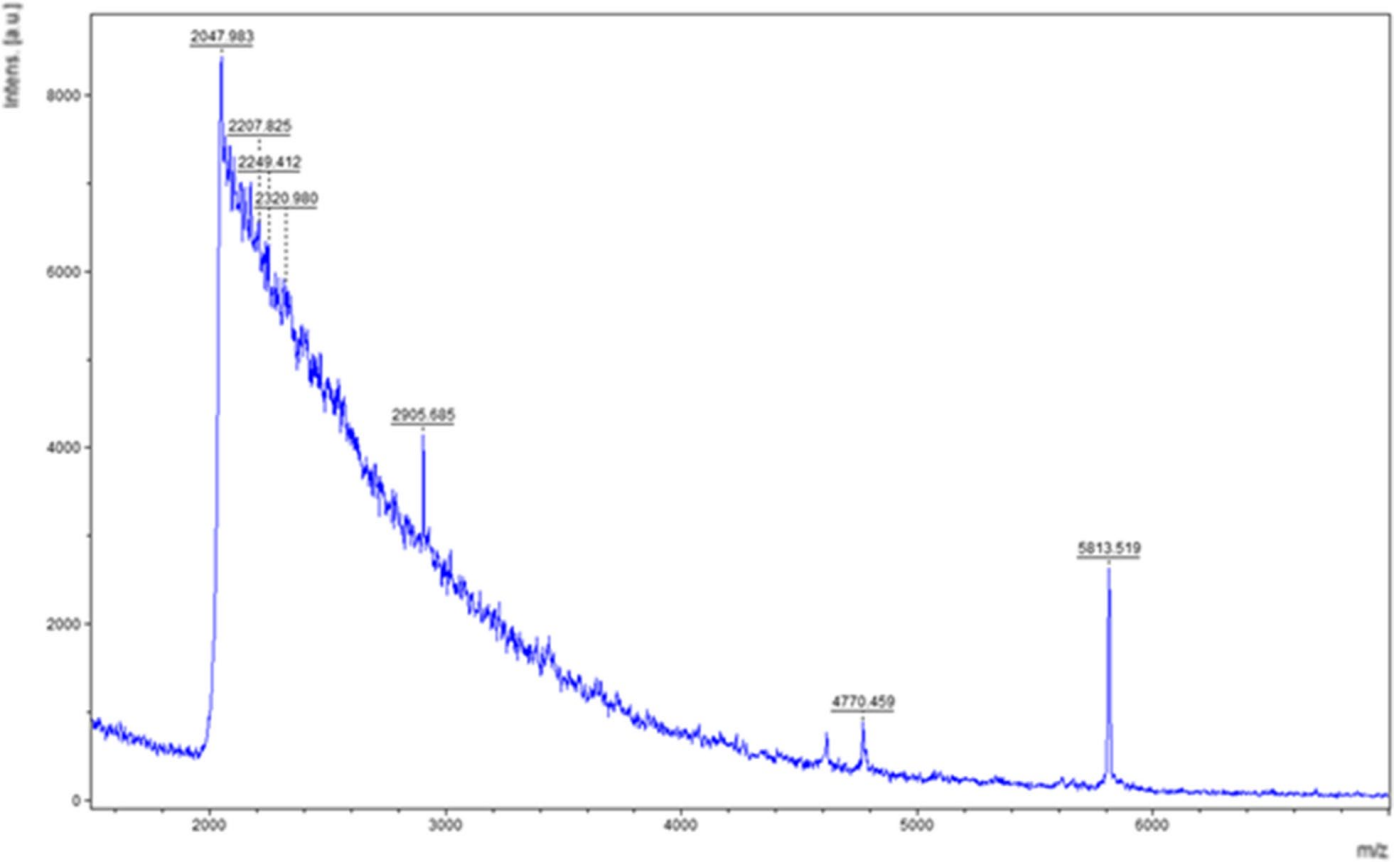
A MALDI-TOF spectrum illustrating the supernatant sample of crude biosynthesised human insulin, which was induced at 1 mM IPTG

The expression of human insulin was conducted by expressing the protein at varying IPTG concentrations, such as 0.1 mM, 0.5 mM, and 1 mM. The standard human insulin and the crude biosynthesised human insulin were detected using MALDI-TOF-MS, as the expected size of human insulin was obtained (approximately 5.8 kDa). Figure 2 shows an example of the MALDI-TOF spectrum, illustrating the supernatant sample of crude biosynthesised human insulin, which was induced at 1 mM IPTG. The results were confirmed using commercial human insulin as the sample using MALDI-TOF (Additional file 1: Figure S2) and LC–MS (Additional file 1: Figure S3). The LC–MS spectra detected the [M+4H]^4+^, [M+5H]^5+^, [M+6H]^6+,^ and M+7H]^7+^ charged states of the human insulin protein in all IPTG induced samples (Additional file 1: Figure S3).

Human insulin was detected in all the intracellular and inclusion body fractions under all IPTG induction conditions. Table 1 shows that 0.1 mM IPTG induced the optimum yield for recovering human insulin using *E. coli* BL21 DE3-pET21b-hPin expression system, according to the standard curve using the commercially available standard human insulin (Additional file 1: Figure S4). Most of the induced human insulin was in the intracellular (soluble) fraction, with 520.92 mg/L of human insulin received in the intracellular fraction for 0.1 mM IPTG induction; compared to 393.81 mg/L and 210.83 mg/L under 0.5 mM and 1.0 mM IPTG, respectively.

**Table 1:** The concentrations of human insulin obtained for the IPTG induction calculated based on commercial human insulin

IPTG induced sample	Concentration (mg/L) (intracellular)	Concentration (mg/L) (inclusion body)
1 mM	210.83	65.45
0.5 mM	393.81	64.20
0.1 mM	520.92	107.82

### Protein sequencing of induced biosynthesised human insulin

**Fig. 3:**
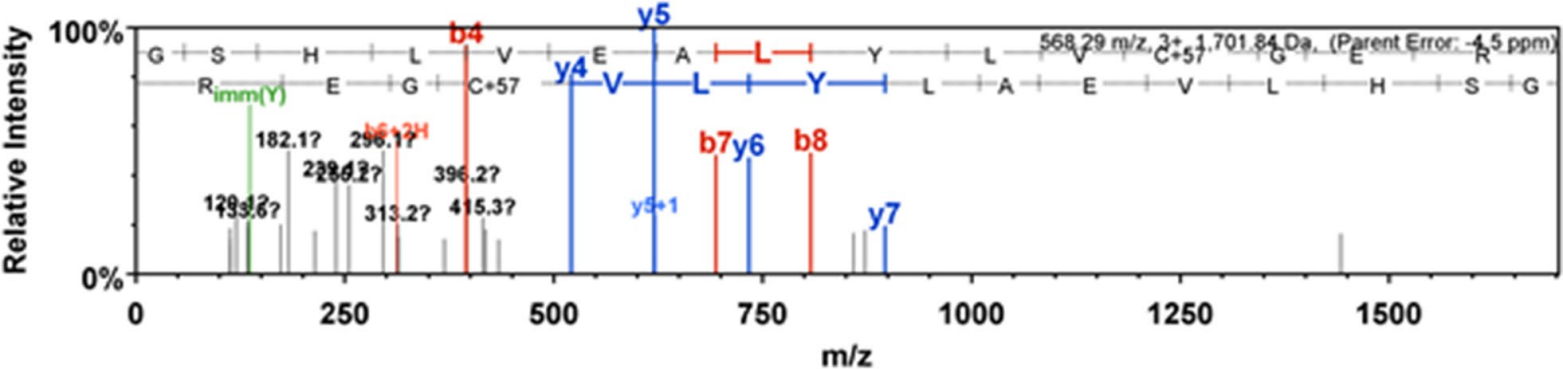
A peptide spectrum illustrating the protein sequence of crude biosynthesised human insulin, which was a 100% match to the human insulin sequence derived from the Scaffold 1.4.4 software

The crude biosynthesised human insulin protein was sequenced. The standard human insulin was set as a positive control. The protein sequencing results confirmed that the biosynthesised protein was human insulin (Fig. 3; Additional file 1: Table S1). After a concatenated database search, the fragmented biosynthesised human insulin peptide sequence was found to have a 100% similarity match to that of the human insulin sequence from the protein database.

### Biological activity of human insulin

**Fig. 4:**
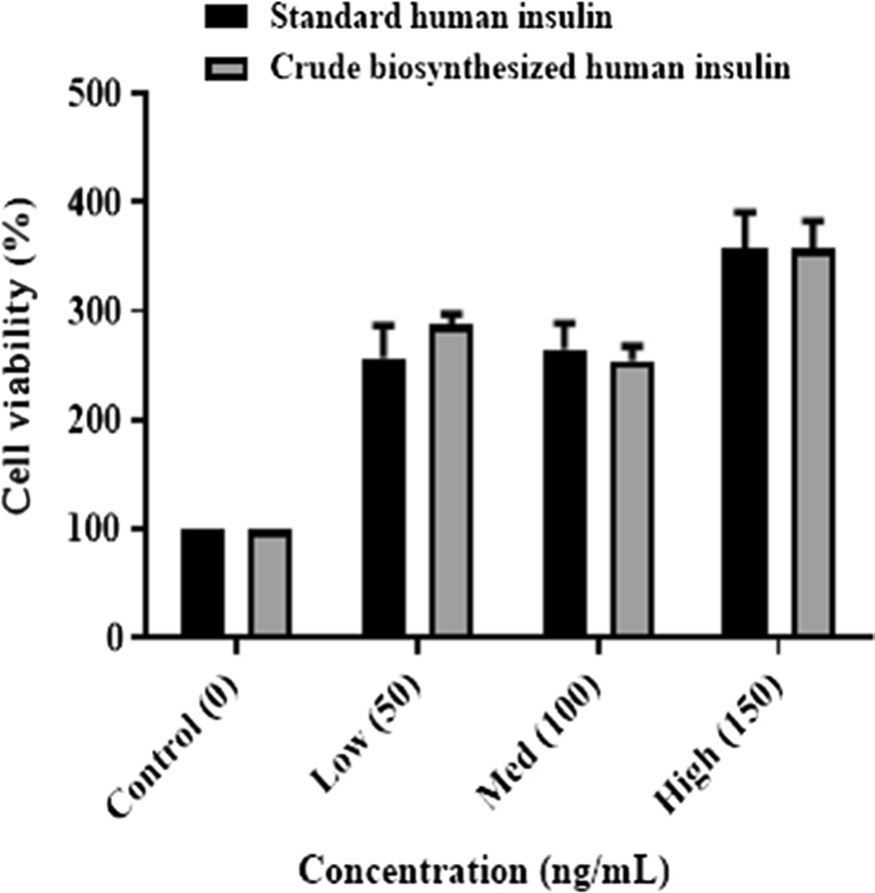
A graph illustrating the cell viability of HepG2 cells, under hyperglycaemic conditions for standard human insulin and crude biosynthesised human insulin at low (50 ng/mL), medium (100 ng/mL) and high (150 ng/mL) MTT concentrations (p-value < 0.0001)

The biological activity was tested using a MTT assay in vitro, using the HepG2 cell line under hyperglycaemic conditions (Fig. 4). In the MTT assay, three concentrations were used of the standard human insulin and crude biosynthesised human insulin: low (50 μg/mL), medium (100 μg/mL) and high (150 μg/mL). The results revealed that, for the low treatment the crude biosynthesised human insulin displayed higher cell viability than the standard human insulin; whereas the medium and high concentrations exhibited similar cell viability to that of the standard human insulin. The results were statistically significant with a calculated probability (p-value) of < 0.0001.

## Discussion

This study developed a novel and efficient biosynthetic laboratory-scale method of human insulin production using an innovative recombinant cloning strategy, pET21b-hPin vector; and an *E. coli* BL21 (DE3) expression system was employed using proinsulin (Fig. 2). Insulin can be biosynthesised using two methods. The first method is when the A and B chains of insulin are cloned separately, isolated, and subsequently purified as S-sulfonate derivatives. The chains are thereafter combined to form insulin, which is then purified (Kroef et al. 1989). The second approach involves the use of proinsulin, which is the preferred method of biosynthesising insulin, since a single fermentation is conducted, as opposed to in the first method of insulin production (Kroef et al. 1989). In terms of costs associated with affinity tag removal on a large scale, the current project was based on the biosynthesis and analysis of the untagged form of human insulin (Norouzi et al. 2016; Young et al. 2012).

This study used a novel PCR-based strategy in the cloning of human insulin. The human insulin gene was amplified and confirmed using PCR (Additional file 1: Figure S1). Subsequently, the pET21b-hPin plasmid was transformed into an expression host BL21 (DE3). The production of biosynthesised human insulin was validated using MALDI-TOF-MS, LC–MS, and protein sequencing (Fig. 3). Commercial human insulin was used as a positive control.

Mention is made in the literature of the human insulin protein being expressed at 1 mM IPTG (Redwan et al. 2007). In this study, the human insulin protein was initially induced with 1 mM IPTG at 37 °C. Thereafter, the optimisation of the expression of human insulin was conducted, where the IPTG concentration was varied. The samples were detected using MALDI-TOF-MS (Fig. 2) and LC–MS (Additional file 1: Figure S3). A standard curve derived from various sample concentrations of standard human insulin was generated using MALDI-TOF-MS to extrapolate the respective protein concentrations from the biosynthesised human insulin supernatant and pellet fractions. The concentrations of the protein found in the intracellular fractions (soluble fractions) were 520.92 mg/L, 393.81 mg/L and 210.83 mg/L; and in the pellets (inclusion body/insoluble fractions) they were 107.82 mg/L, 64.20 mg/L and 65.45 mg/L for the respective IPTG concentrations of 0.1 mM, 0.5 mM and 1 mM (Table 1). The optimum condition for the induction of human insulin was 0.1 mM IPTG.

This study optimised human insulin protein expression, where the human insulin protein was expressed in the soluble (intracellular) fraction resulting in the elimination of the use of chaotropic agents. The use of chaotropic agents, such as urea and guanidine hydrochloride, can result in complete secondary structure denaturation (Singh et al. 2015). The use of guanidine hydrochloride and urea to solubilise protein results in a low yield of the bioactive therapeutic protein (Upadhyay et al. 2016). The proinsulin was produced in inclusion bodies at a 10% level in *E*. *coli* with a yield of 1.3 mg/L of insulin (Redwan et al. 2007). Solubilisation and refolding can often lead to poor recovery of the protein of the desired bioactive protein (Singh et al. 2015). This study had a yield of approximately 520.92 (mg/L) of human insulin located in the intracellular (soluble) fraction, which is higher than in previous reports (Redwan et al. 2007).

Researchers have found that protein expression conducted at a high inducer concentration and temperature resulted in the expression of the biosynthesised protein at an increased translational rate and can eventually result in the formation of inclusion bodies (Carrió and Villaverde 2005). The formation of inclusion bodies in bacterial expression hosts such as *E*. *coli* presents challenges in the recovery process of bioactive proteins (Upadhyay et al. 2016). The extraction of recombinant biosynthesised proteins from inclusion bodies usually results in low yields of the bioactive protein, and this process is laborious (Singh et al. 2015). Inclusion bodies require downstream processing such as isolation from the cell, solubilisation, purification, and refolding of the protein (Redwan et al. 2007; Singh et al. 2015; Zieliński et al. 2019). In this study, the protein was expressed mainly in the soluble fraction; therefore, the inclusion body recovery and renaturation of the protein steps were eliminated, which decreased the expenses associated with these steps. Therefore, the method of human insulin biosynthesis employed in this study is a more efficient process than the current methods (Zieliński et al. 2019).

The proinsulin cloning strategy uses the nucleotide sequence encoding for human proinsulin, which consisted of A, B, and C chains (Chance et al. 1999). In the above strategy, proinsulin was cloned, and the C-peptide was cleaved using *Achromobacter lyticus* protease, carboxypeptidase B, and trypsin (Baeshen et al. 2014; Mollerup et al. 2009; Morihara et al. 1980). *Achromobacter lyticus* protease (Morihara et al. 1980) is a lysine-specific enzyme which can enzymatically convert proinsulin to insulin; it has an advantage over trypsin digestion as it avoids non-specific cleavage after the arginine at position B22 (Mollerup et al. 2009). The C-peptide bond was enzymatically cleaved by the utilisation of cyanogen bromide; this was followed by protein folding and the formation of disulphide bonds (Frank and Chance 1983). The enzymes that are normally used in the C-peptide cleavage, such as carboxypeptidase B, are costly (Redwan et al. 2007). This ground-breaking study eliminated the expensive digestion of the C-peptide, which occurred as a result of autocatalytic cleavage (Gooch 2011).

The human insulin that was biosynthesised in this study was detected and verified using MALDI-TOF-MS, LC–MS, and protein sequencing. Standard human insulin served as a positive control for the protein sequencing of human insulin with a 90% similarity to the human insulin sequence from the protein database (Additional file 1: Figure S5). The crude biosynthesised human insulin protein produced in this study was verified using protein sequencing. After a concatenated peptide database search, the crude biosynthesised human insulin was unequivocally proven to be human insulin with a 100% similarity (Fig. 3). Therefore, this study successfully biosynthesised and optimised the expression of human insulin in *E*. *coli*.

This study employed an in vitro MTT assay to determine the biological activity. The MTT assay is used to measure the viability of metabolically active cells based on their ability to produce reducing equivalents (Carrió and Villaverde 2005; Morihara et al. 1980). The cell uptake of MTT by a protein facilitated mechanism or by endocytosis leads to the reduction of MTT. This yields a purple formazan product, which is impermeable to cell membranes and results in its accumulation within living cells (Hansen and Bross 2010; Maioli et al. 2009; Mosmann 1983). The solubilisation of the cells results in the release of the purple product, which is detected colorimetrically (Maioli et al. 2009). The cells’ respiratory chain (Slater et al. 1963) and other electron transport systems (Liu et al. 1997) cause the reduction of MTT. The reduction of MTT by living cells is an indication of mitochondrial activity, which therefore serves as a measure of cell viability (Maioli et al. 2009). Insulin is involved in the regulation of glucose uptake into muscle and fat cells (Drejer 1992). Insulin works within seconds to activate the tyrosine kinase receptor, ion transport systems, and stimulation of glucose (Drejer 1992). Therefore, the glucose utilisation by the HepG2 cells results in an increase of cellular respiration and mitochondrial activity, resulting in an increase in cell viability. The biological activity was tested in this study using a MTT assay in vitro, using a HepG2 cell line with normoglycaemic (5.5 mM) and hyperglycaemic (25 mM) conditions (Chen et al. 2006). The MTT assay exhibited increased cell viability in the hyperglycaemic treatments. There were three treatment concentrations of standard human insulin and crude biosynthesised human insulin: low (50 ng/mL), medium (100 ng/mL), and high (150 ng/mL). The analysis was conducted on standard human insulin and biosynthesised human insulin. The standard human insulin served as a positive control. The cell viability of the crude biosynthesised human insulin showed (288.42% ± 9.04%) low, (254.57% ± 12.96%) medium and (358.70% ± 24.17%) high concentrations, in comparison to standard human insulin which had (257.37% ± 29.65%) low, (265.23% ± 23.61%) medium and (358.07% ± 33.10%) high concentrations. In the low concentration, the crude biosynthesised human insulin displayed higher cell viability than the standard human insulin; whereas the medium and high concentrations exhibited similar cell viability to that of the standard human insulin. As such, the efficacy was determined to be the same as the standard human insulin. Therefore, we can conclude that the novel method employed in this study is an efficient and effective method of human insulin production.

The biological activity test conducted in this study revealed that the crude biosynthesised human insulin displays similar efficacy to that of the commercially available standard human insulin. This study successfully provided a novel method for the biosynthesis of human insulin since it employed the untagged pET21b expression vector in *E*. *coli*. The elimination of affinity tags has implications with regards to protein purification, as well as the reduction in downstream processing costs, such as glutathione *S*-transferase and histidine tag removal. In addition, this method also eliminated the tedious protein enzymatic cleavage of the C-peptide, the recovery of human insulin from inclusion bodies, and human insulin renaturation steps; thereby making this process less labour intensive. This study was conducted on a laboratory-scale; however, it has the potential to be up-scaled industrially. Future studies can focus on finding a greener and more affordable purification method for human insulin.

## Supplementary information

**Additional file 1. Figure S1a.** An image depicting a purified PCR product of human proinsulin gene. Lane 1 contains the one kb molecular weight marker, and lane 2 contains the purified *Bam*HI, and an *Xho*I ended PCR product of human proinsulin gene (345 bp). **b.** An image displaying colony PCR products of positive transformants obtained from clones that contain the pET21b-hPin vector. Lane 1 contains the one kb molecular weight marker, and lanes 2-5 contain colony PCR amplicons (345 bp). **Figure S2.** An image depicting a MALDI-TOF spectrum of standard human insulin as a positive control. **Figure S3.** A LC–MS chromatogram illustrating 1 mM IPTG induced biosynthesised human insulin sample. **Figure S4.** A MALDI-TOF standard curve of the human insulin standard at 0 ng, 10 ng, 50 ng, 100 ng, and 1000 ng. **Figure S5.** A peptide spectrum illustrating the protein sequence of standard human insulin, which was 90% similar to the human insulin sequence derived from Scaffold 1.4.4 software. **Table S1.** Peptide fragmentation table of crude biosynthesised human insulin, which yielded a 100% match to human insulin in the protein database.

## Data Availability

The data generated during this study are included in the published article (and its online supplementary file). Additional information regarding the data generated from the current study is available from the corresponding author upon request.

## Abbreviations

*E*. *coli*: *Escherichia coli*
IU: International units
aa: Amino acid
DNA: Deoxyribonucleic acid
RP-HPLC: Reversed phase-high performance liquid chromatography
PCR: Polymerase chain reaction
USA: United States of America
rpm: Revs per minute
OD_600_: Optical density at a wavelength of 600 nm
LB: Luria–Bertani
IPTG: Isopropyl β-d thiogalactoside
kDa: Kilo-Dalton
MALDI-TOF-MS: Matrix-assisted laser desorption ionization-time of flight-mass spectrometry
kV: Kilovolts
Da: Dalton
nm: Nanometres
CHCA or HCCA: Alpha-cyano-4-hydroxycinnamic acid
LC–MS: Liquid chromatography–mass spectrometry
cRAP: Contaminant protein database
MTT: 3-(4, 5-Dimethylthiazol-2-yl)-2, 5-diphenyl tetrazolium bromide
HepG2: Hepatocellular carcinoma
PBS: Phosphate-buffered saline
DMSO: Dimethyl sulfoxide
OD: Optical densities
ANOVA: Analysis of variance
p-value: Calculated probability
