# Supplementary material for: A novel and more efficient biosynthesis approach for human insulin production in Escherichia coli (E. coli)
Source: AMB Express. 2020 Mar 10;10:43. doi: 10.1186/s13568-020-00969-w (PMC7062966; doi:10.1186/s13568-020-00969-w)
Supplement: Supplementary file 1 — Additional file 1. Figure S1a. An image depicting a purified PCR product of human proinsulin gene. Lane 1 contains the one kb molecular weight marker, and lane 2 contains the purified BamHI, and an XhoI ended PCR product of human proinsulin gene (345 bp). b. An image displaying colony PCR products of positive transformants obtained from clones that contain the pET21b-hPin vector. Lane 1 contains the one kb molecular weight marker, and lanes 2-5 contain colony PCR amplicons (345 bp). Figure S2. An image depicting a MALDI-TOF spectrum of standard human insulin as a positive control. Figure S3. A LC–MS chromatogram illustrating 1 mM IPTG induced biosynthesised human insulin sample. Figure S4. A MALDI-TOF standard curve of the human insulin standard at 0 ng, 10 ng, 50 ng, 100 ng, and 1000 ng. Figure S5. A peptide spectrum illustrating the protein sequence of standard human insulin, which was 90% similar to the human insulin sequence derived from Scaffold 1.4.4 software. Table S1. Peptide fragmentation table of crude biosynthesised human insulin, which yielded a 100% match to human insulin in the protein database. [file 13568_2020_969_MOESM1_ESM.pdf]

Journal: Applied Microbiology and Biotechnology Express.

A novel and more efficient biosynthesis approach for human insulin production in *Escherichia coli (E. coli)*.

Kamini Govender[1], Tricia Naicker[1], Johnson Lin[2], Sooraj Baijnath[1], Anil Amichund Chuturgoon[3], Naeem Sheik Abdul[3], Taskeen Docrat[3], Hendrik Gerhardus Kruger[1*] and Thavendran Govender[4*]

[1]Catalysis and Peptide Research Unit, School of Health Sciences, University of KwaZulu-Natal, Durban, South Africa

[2]School of Life Sciences, University of KwaZulu-Natal, Durban, South Africa

[3]School of Laboratory Medicine and Medical Sciences, College of Health Sciences, University of KwaZulu-Natal, Durban, South Africa

[4] Department of Chemistry, University of Zululand, Private Bag X1001, KwaDlangezwa 3886, South Africa

*Corresponding authors: kruger@ukzn.ac.za, Orchid ID: 0000-0003-0606-2053 and govendert@unizulu.ac.za

Catalysis and Peptide Research Unit (CPRU)

E-block, 6th Floor, Room E1-06-016

University of KwaZulu-Natal, Westville Campus, South Africa

Contact number: +27312601845

The supplementary information contains five figures and one table.

LIST OF FIGURES AND TABLES

Figure S1a: An image depicting a purified PCR product of human proinsulin gene. Lane 1 contains the one kb molecular weight marker, and lane 2 contains the purified *Bam*HI, and an *Xho*I ended PCR product of human proinsulin gene (345 bp). 1

Figure S1b: An image displaying colony PCR products of positive transformants obtained from clones that contain the pET21b-hPin vector. Lane 1 contains the one kb molecular weight marker, and lanes 2-5 contain colony PCR amplicons (345 bp). 1

Figure S2: An image depicting a MALDI-TOF spectrum of standard human insulin as a positive control. 2

Figure S3: A LC-MS chromatogram illustrating 1 mM IPTG induced biosynthesised human insulin sample. 2

Figure S4: A MALDI-TOF standard curve of the human insulin standard at 0 ng, 10 ng, 50 ng, 100 ng, and 1000 ng. 3

Figure S5: A peptide spectrum illustrating the protein sequence of standard human insulin, which was 90% similar to the human insulin sequence derived from Scaffold 1.4.4 software. 3

Table S1: Peptide fragmentation table of crude biosynthesised human insulin, which yielded a 100% match to human insulin in the protein database. 3

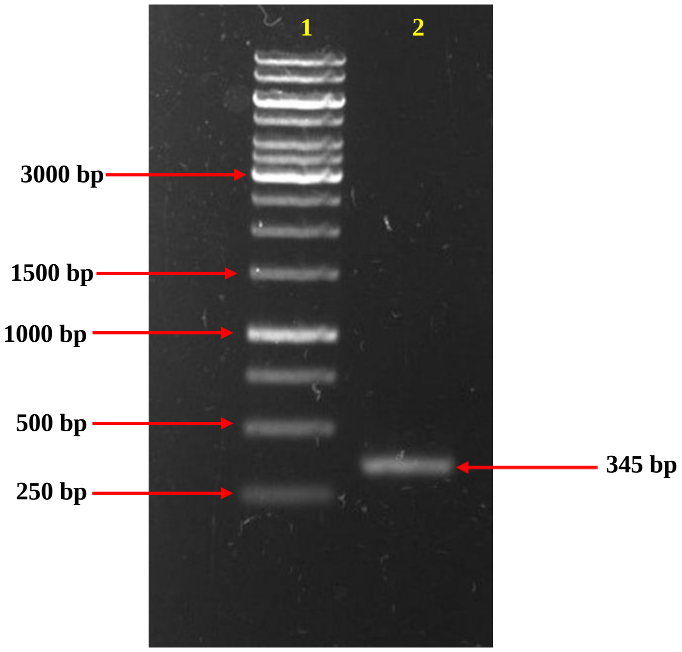

Figure S1a: An image depicting a purified PCR product of human proinsulin gene. Lane 1 contains the one kb molecular weight marker, and lane 2 contains the purified *Bam*HI, and an *Xho*I ended PCR product of human proinsulin gene (345 bp).

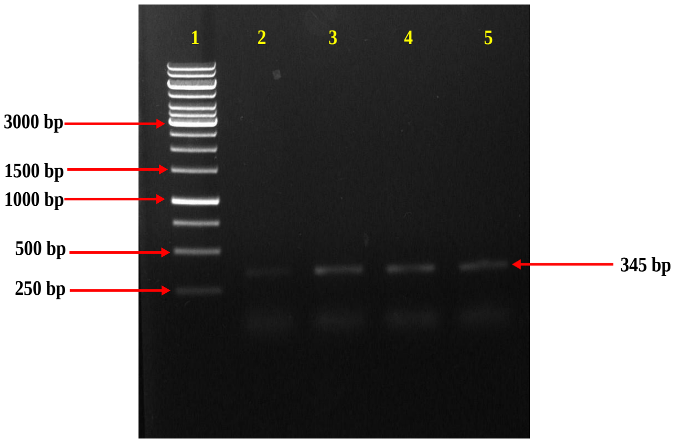

Figure S1b: An image displaying colony PCR products of positive transformants obtained from clones that contain the pET21b-hPin vector. Lane 1 contains the one kb molecular weight marker, and lanes 2-5 contain colony PCR amplicons (345 bp).

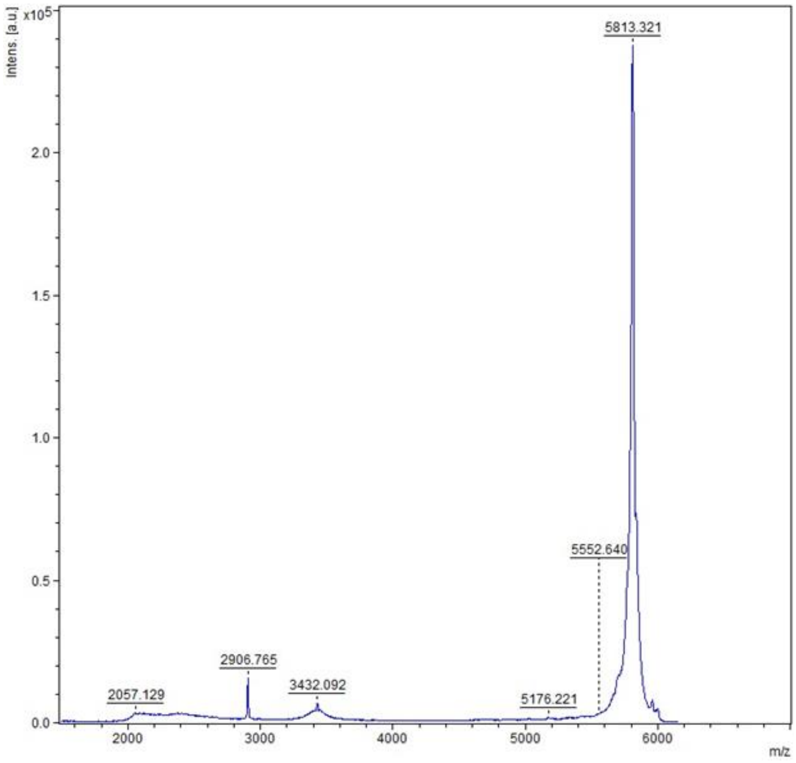

Figure S2: An image depicting a MALDI-TOF spectrum of standard human insulin as a positive control.

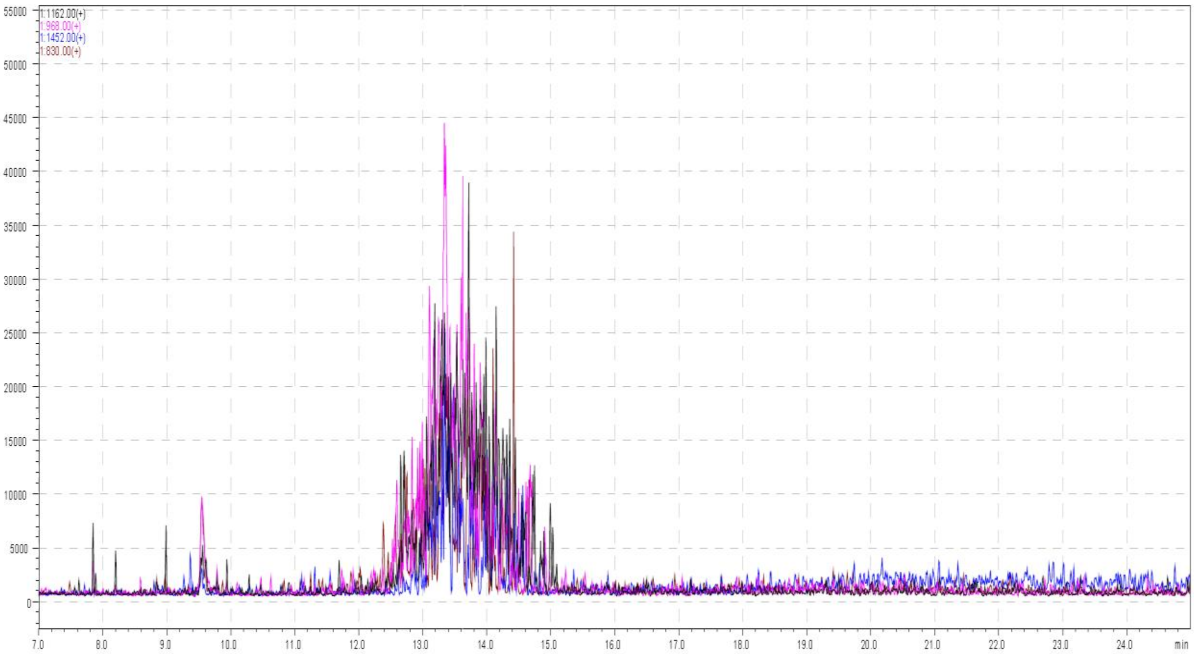

Figure S3: A LC-MS chromatogram illustrating 1 mM IPTG induced biosynthesised human insulin sample.

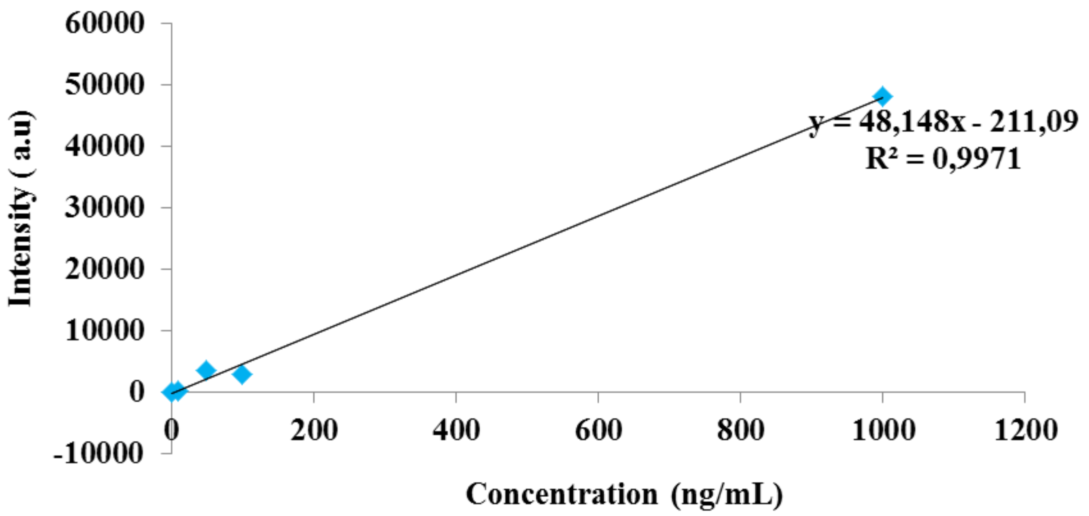

Figure S4: A MALDI-TOF standard curve of the human insulin standard at 0 ng, 10 ng, 50 ng, 100 ng, and 1000 ng.

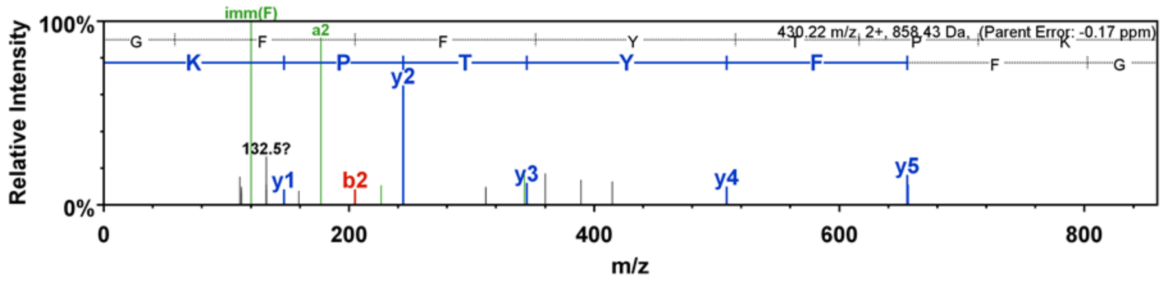

Figure S5: A peptide spectrum illustrating the protein sequence of standard human insulin, which was 90% similar to the human insulin sequence derived from Scaffold 1.4.4 software.

Table S1: Peptide fragmentation table of crude biosynthesised human insulin, which yielded a 100% match to human insulin in the protein database.

B	B Ions	B + 2H	B-NH3	B-H2O	AA	Y Ions	Y + 2H	Y-NH3	Y-H2O	Y
1	58.0	29.5			G	1702.6	851.9	1685.8	1684.8	15
2	145.1	73.0		127.1	S	1645.8	823.4	1628.8	1627.8	14
3	282.1	141.6		264.1	H	1558.8	779.9	1541.8	1540.8	13
4	395.2	198.1		377.2	L	1421.7	711.4	1404.7	1403.7	12
5	494.3	247.6		476.3	V	1308.7	654.8	1291.6	1290.7	11
6	623.3	312.2		605.3	E	1209.6	605.3	1192.6	1191.6	10
7	694.4	347.7		676.3	A	1080.6	540.8	1063.5	1062.5	9
8	807.4	404.2		789.4	L	1009.5	505.3	992.5	991.5	8
9	970.5	485.8		952.5	Y	896.4	448.7	879.4	878.4	7
10	1083.6	542.3		1065.6	L	733.4	367.2	716.3	715.4	6
11	1182.7	591.8		1164.6	V	620.3	310.6	603.3	602.3	5
12	1342.7	671.8		1324.7	C + 57	521.2	261.1	504.2	503.2	4
13	1399.7	700.4		1381.7	G	361.2	181.1	344.2	343.2	3
14	1528.7	764.9		1510.7	E	304.2	152.6	287.1	286.2	2
15	1702.9	851.9	1685.8	1684.8	R	175.1	88.1	158.1		1
